# Impact of Previous Ankle Injuries on Professional Footballers' Preseason Functional Ankle Assessment

**DOI:** 10.5704/MOJ.2311.011

**Published:** 2023-11

**Authors:** AH Ahmad-Shushami, MS A-Hamid, MH Khair, MO Ali

**Affiliations:** 1 Department of Sports Medicine, Universiti Malaya, Kuala Lumpur, Malaysia; 2 Department of Sports Medicine and Sports Science, Selangor Football Club, Shah Alam, Malaysia

**Keywords:** ankle injury, football, ankle joint, postural stability

## Abstract

**Introduction:**

Football is the most popular sport and is widely played around the globe, with approximately 400 million players in 208 countries. Lower extremity injuries showed the highest incidence, with ankle injuries being the most prevalent after hip and knee injuries. The purpose of this study was to describe the characteristics of the players who reported previous ankle injuries during pre-competition medical assessment (PCMA) during the 2022 seasons of the Malaysian professional club. In addition, the study also investigated the effect of previous injuries on current ankle function.

**Materials and methods:**

This was a retrospective cross-sectional study using secondary data from the preseason PCMA data from a professional club that competed in Malaysia. The ankle range of motion, anterior drawer test, and functional ankle assessments including the Biodex athlete single leg stability test and ankle joint muscle strength were performed during the PCMA.

**Results:**

A total of 45 footballers reported previous history of ankle injuries to the left (n=9), right (n=20), or both ankles (n=16). Footballers with prior ankle injuries exhibited significantly less ankle inversion (p = 0.008) and a larger proportion of positive ADT tests in the injured ankle (x^2^(1, N=90) =7.76, p=0.005) compared to the non-injured side. there was no significant difference in other ankle range of motion, ankle stability index, or ankle muscular strength between previously injured and uninjured ankles.

**Conclusions:**

During preseason screening, half of the footballers in this study reported previous history of ankle injury, putting them at risk of having future ankle injuries. Aside from inversion and the anterior drawer test, no significant differences in range of motion, stability index, or muscle strength were discovered. However, as injury causation is multifactorial, preventive measures should be taken to reduce the risk of injury.

## Introduction

Football is the most popular sports and widely played around the globe with approximately 400 million players in 208 countries^1^. Similar to other sports, it carries a certain risk of injuries. The injury incidence varies depending on the level of competition and player characteristics such as age, gender and skill level^2^.

A meta-analysis of 44 studies found that the incidence of injuries among professional male football players was ten times greater during matches (36 injuries/1000 hours of exposure) than during training (3.7 injuries/1000 hours of exposure). Lower extremity injuries showed the highest incidence rates (6.8 injuries/1000 hours of exposure) when compared to other body parts, with ankle injuries being the most prevalent after hip and knee injuries^3^. It is critical to understand that sports injuries are multifactorial. Although injury can be caused by a single triggering event, it is more often caused by a complex interaction of internal and external risk factors^4^.

In a study conducted among a general clinic-based population, residual symptoms were reported in 72% of the subjects within 6 to 18 months post injury, and 40% had moderate to severe symptoms, namely ankle instability, calf muscle weakness, pain, or swelling^5^. The study also reported, ankle reinjury, immobilisation for more than one week, and limitation in weight bearing for more than 28 days were significant predictors of residual ankle symptoms^5^. Wisthoff *et al* found that subjective scores of ankle function and stability were reduced in the group with lateral ankle sprain in comparison to a group of subjects without injury. The injured group were also found to have greater laxity on the anterior drawer test and a more range of ankle inversion compared to the control group. This findings showed that long term deficits due acute lateral ankle sprain may prolonged up to six months post injury^6^.

A prospective cohort study involving 82 first-time ankle sprains found that 40% of patients developed chronic ankle instability (CAI)^7^ one year after the initial injury^8^. Moreover, 60% of the patients ankle symptoms improved (copers) and recovered one year following intervention^7^.

Several studies have been conducted to identify risk factors of ankle injuries^9-12^. Previous ankle injury is a significant predictor of subsequent ankle sprain in athletes^13^. Poor Y-Balance Test score was found to increase risk of ankle injury in both dominant and nondominant limbs^14^. Additionally, a meta-analysis found that body mass index, slow eccentric inversion strength, fast concentric plantar flexion strength, passive inversion joint position sense, and peroneus brevis reaction time significantly increased the risk of lateral ankle sprain^15^.

Therefore, preseason screening is essential in identifying players who could potentially be at risk of sustaining another injury during the regular season. Moreover, optimal ankle rehabilitation program is important to prevent injury recurrence. The purpose of this study was to describe the characteristics of the players who reported previous ankle injuries during pre-competition medical assessment (PCMA) during the 2022 seasons of the Malaysian Super League, Malaysian Premier League, President Cup, and Youth Cup. In addition, the study also investigated the effect of previous injuries on current ankle function. This study will help the team develop an effective injury prevention programme and subsequently safeguard the players' health and wellbeing.

## Materials and Methods

This is a retrospective cross-sectional study using secondary data from the preseason Pre-Competition Medical Assessment (PCMA). The study was approved by the Medical Research Ethics Committee, University Malaya Medical Centre (ID NO 2022216-11002). The PCMA data from a man professional club competed in the 2022 Malaysian Super League, Malaysian Premier League, President Cup, and Youth Cup were used in this study. The medical records of all football players who have registered with the club and fully completed the PCMA were retrieved. Incomplete medical records were excluded from analysis.

The pre-competition medical evaluation (PCMA) was made compulsories for all professional football players competing in any tournament organised by the Football Association of Malaysia. The purpose of the PCMA is to ensure safe sports participation. During the PCMA, players' sociodemographic information and medical information (including previous injuries) were obtained. Injury was defined as any physical complaint sustained by a player that results from a football match or football training, irrespective of the need for medical attention or time loss from football activities^16^. A recurrent injury was defined as an injury of the same type and at the same site as an index injury that occurs after a player’s return to full participation from the index injury^16^.

A complete medical assessment was performed at Sports Medicine Clinic, Universiti Malaya Medical Centre from January to February 2022. The PCMA mainly focused on musculoskeletal and cardiovascular screening. Apart from that, general assessment of other systems such as respiratory, abdominal, and endocrine were also evaluated.

During the PCMA, the ankle range of motion including plantarfexion, dorsiflexion, inversion and eversion were assessed using a standard goniometer based on technique described by previous authors^17,18^. The state of the ankle ligaments were assessed using the anterior drawer test as described by Croy and Saliba^19^.

Two functional ankle assessments were performed in this study, namely the ankle muscle strength and ankle balance test. The ankle muscle strength was assessed using a hand-held dynamometer (HHD) Commander JT-CM300 [Jtech Medical Industry, UT] according to previous studies^20,21^. Ankle plantarflexion and dorsiflexion muscle strengths were assessed with athlete positioned in supine and the ankle in a neutral position^21^. Ankle inversion strength measurement, the HHD transducer was positioned at the medial border of the forefoot directly beneath the first metatarsal head. For assessment of foot eversion strength, the transducer of the HHD was positioned at the lateral forefoot border, right beneath the fifth metatarsal head^20^. In addition, athletic single-leg stability test was performed with the Balance System^TM^ [Biodex Medical System Inc, NY] to assess dynamic balance as described in other studies^22-24^.

Continuous variable data distribution was examined using the central tendency and dispersion methods. Data were presented as mean (SD) or median (IQR) accordingly. Categorical data, on the other hand, was presented as frequency and percentages.

For continuous data with normal distribution, paired t-test was performed to compare findings between footballers with and without previous ankle injury. The Wilcoxon signed rank test was performed to compare the difference between groups with not normally distributed data.

For categorical data, crosstabulation was performed to analyse association between previous history of ankle injury the anterior drawer test, chi-square test was performed to determine significance of the relationship. A P value of 0.05 was considered statistically significant. All statistical analysis was performed using SPSS statistical software [Version 27; IBM Corp].

A stepwise logistic regression analysis was conducted to identify the predictors of ankle injuries. Variables <0.25 on univariate testing were included in the multivariate logistic regression model, as recommended by previous researchers^25^. Adjusted odds ratios (ORs) and 95% confidence intervals (CIs) of ORs were calculated, with the significance level set at p<0.05 (25).

## Results

A total of 94 footballers’ pre-competition medical assessment (PCMA) records during the 2022 preseason were reviewed. However, four footballers did not respond to questions about their previous ankle injuries, hence their information were not further analysed. The mean age of the football players in this study was 21.2±3.9 years. Forty-five footballers reported previous history of ankle injuries involving the left (n=9), right (n=20) or both ankles (n=16). The characteristics of players with and without a history of prior injury are shown in Table I. There was no significant difference in the players’ characteristics between the two groups.

**Table I:** Descriptive characteristic for players with and without history of previous ankle injury.

Variables (@)	No history of ankle injury (n=45)	Previous history of ankle injury (n=45)	p-value
Age* (years)	20.0 (4.5)	20.0 (2.0)	0.82
Body weight (kg)	67.4 (7.7)	67.7 (8.2)	0.86
SMM* (kg)	32.3 (6.5)	35.1 (11.9)	0.48
Height (cm)	172.2 (7.7)	172.6 (7.1)	0.82
BMI (kg/m^2^)	22.7 (1.6)	22.6 (2.1)	0.97
Dominant leg (%)			
Right	34(75.6)	35(77.8)	0.80
Left	11 (24.2)	10 (22.2)	
Position held			
Goalkeeper	5 (11.1)	6 (13.3)	0.56
Defender	13 (28.9)	15 (33.3)	
Midfielder	12 (26.7)	15 (33.3)	
Striker	15 (33.3)	9 (20.0)	

Abbreviations – SMM: skeletal muscle mass, BMI: body mass index Notes: *Data are presented as median (IQR) for abnormally distributed data @ (4 missing data)

The functional assessments of injured and non-injured ankles are presented in Table II. Footballers with previous ankle injuries had significantly less ankle inversion in the injured ankle compared to the non-injured side (p=0.008).

**Table II:** Ankle assessment of players with history of previous ankle injury.

Variables (n=45)	Previously injured site	Uninjured site	p-value
Range of Motion			
Dorsiflexion	10.0 (6.0)	12.0 (7.0)	0.81
Plantarflexionª	54.4 (9.3)	53.9 (9.3)	0.71
Inversion	35.0 (10.0)	40.0 (12.8)	0.008^b^
Eversion	15.0 (6.5)	15.0 (8.0)	0.32
Isometric Muscle Strength Testing			
Dorsiflexionª	177.8 (46.6)	174.2 (39.4)	0.35
Plantarflexionª	191.1 (56.2)	185.2 (53.3)	0.11
Inversion	136.0 (52.0)	132.0 (51.0)	0.84
Eversion	132.0 (46.0)	129 (44.0)	0.87
Athlete single leg stability test			
Overall stability index	1.3 (0.5)	1.3 (0.7)	0.23
Anterior-Posterior stability index	0.9 (0.3)	0.9 (0.4)	0.59
Medial lateral stability index	0.8 (0.3)	0.9 (0.4)	0.48

Notes: ^a^ data are presented as mean (SD) and paired t-test was performed for normally distributed data ^b^ statistically significant (p value <0.05)

A chi-square test of independence was performed to assess relationship between previous history of ankle injury and the ADT result. There was a significant relationship between the two variables, X^2^(1,N=90) =7.76, p=0.005. Higher proportion of positive ADT test were found among footballers with previous history of ankle injury. On further analysis, there was no significant difference in other ankle range of motion, ankle stability index, or ankle muscular strength between previously injured and uninjured ankles.

Only three variables on univariate analysis found p<0.25 and were included in the logistic regression analysis (inversion ROM, plantarflexion strength and overall stability index). However, no statistically significant predictors (based on these three variables) were identified by logistic regression analysis.

## Discussion

The preseason Pre-Competition Medical Assessment (PCMA) is an important component to identify possible risk factors that may increase the risk of injuries occurrence during the competitive football season. Furthermore, if any dysfunctional parameters are identified during preseason functional evaluation, preventive measures could be taken to minimise the risk. The aim of this study was to describe the characteristics of the players who reported previous ankle injuries during pre-competition medical assessment.

Our findings indicate that 50% of the professional club footballers reported previous history of ankle injuries on one or both ankles. Previous studies also reported 43 to 78.7% of their study participants reported previous history of ankle injuries^12–14,26^. Several studies have found that a previous history of ankle injury is a strong predictor of injury occurrence. According to a study by Pourgharib et al among elite football and basketball players, past episodes of acute and recurrent ankle sprains were the most powerful predictors of future ankle injury^13^. In another study of intrinsic risk factors for ankle sprain among active university students, subjects with history of previous sprain were twice as likely to sustain a subsequent ankle injury^26^. Therefore, it is a crucial information for the team that half of the players are at risk of getting another injury during the competitive season and appropriate measure should be taken to prevent the injury recurrence.

A comprehensive assessment of both passive and active ankle joint range of motion is particularly important in managing ankle injuries^27^. Hubbard and Cordova found increased ankle inversion rotation in the injured ankle at the 8th week post injury^28^. Moreover, Wisthoff et al found that ankle inversion was significantly greater six months after injury among 108 volunteers comprised of recreational or competitive university student-athletes^6^. Both studies compared ankle inversion range of movement between individuals who had ankle injury with non-injured controls. Contrary to our findings, there was significantly less ankle inversion on the previously injured site among footballers with history ankle injuries (p<0.05). In our study, we compared the previously injured ankle with the uninjured sides of the same individual. We postulate lesser ankle inversion observed in the current study could be associated with soft tissue healing including contracture of scar tissue^7,27^,. Improvement in ankle inversion among the injured group were also reported by Wisthoff who found reduced ankle inversion by at two to four weeks post injury^6^. Moreover, Liu *et al* also found that there was no significant increment in inversion and ligament laxity despite recurrent sprains among collegiate athletes^29^.

Other than inversion, individuals with restricted dorsiflexion ROM have been shown to have an increased risk of sustaining ankle sprains when compared to individuals with normal dorsiflexion ROM^30^. In our study, there was no difference in ankle dorsiflexion between sites with and without previous injury.

Denegar et al investigated 12 athletes with a history of ankle sprain who had already returned to sports and found that there was no difference in ankle dorsiflexion ROM compared to uninjured sites^31^. A study by Wisthoff et al also found that there was no difference in ankle dorsiflexion ROM at six months post injury6. We postulate that our study population of professional footballers who sustained ankle injuries underwent optimised rehabilitation programmes since there were no range of motion deficits. To ensure optimise medical care, it is important to identify athletes with reduced range of motion, particularly ankle dorsiflexion, during preseason so that early preventive measures can be applied.

Significantly higher proportion of participants with previous ankle injuries had positive anterior drawer test than footballers without history of ankle injury. These findings suggest that a previous history of injury contributed to persistent ankle laxity among footballers. Interestingly, there was no difference in ankle stability index between the two groups (p-value >0.05). These findings demonstrated that persistent ankle laxity not necessarily leads to ankle instability. In a study by Lohrer et al among 41 recreational athletes, the authors could not establish a clear relationship between mechanical laxity and functional ankle^32^.

In their study, among the less symptomatic group, subjects with mechanically stable ankles demonstrated no difference in daily and sports functional scores compared to subjects with mechanically unstable ankles^32^. However, the author concluded that both measurement should be taken into account in clinical assessment^32^. Fousekis et al discovered in a study involving 100 professional football players that ankle laxity is not a significant risk factor for ankle injury^10^. Another study conducted among football and basketball players also could not establish a relationship between ankle joint laxity and the occurrence of ankle injuries^13^. In contrast, Beynon *et al* found that men with increased talar tilt are at greater risk of incurring ankle injuries^11^.

Furthermore, based on the updated CAI model, the causative factors of instability are not just limited to pathomechanical impairments, including pathologic laxity, but also involved a complex interaction with sensory-perceptual impairments and motor behavioural impairments^33^. Therefore, assessment of ankle laxity is an important component of injury risk factors during preseason, but the findings must be interpreted in combination with other functional ankle assessments.

Poor balance has been identified as a predictor of an athlete's susceptibility to ankle sprain. A prospective study among 210 football players showed that injured participants had a significantly poorer balance score on the wobble board compared to uninjured participants^34^. A systematic review showed that slow eccentric inversion strength and fast concentric plantar flexion strength were associated with an increased risk of incurring lateral ankle sprain^15^. In our investigation, there was no difference in the stability index score between footballers with and without a history of prior injury. Since there was no difference in the isometric strength of the ankle in plantarflexion, dorsiflexion, and eversion between footballers. We postulate that footballers with a history of previous ankle injury in this study have recovered functionally and become ‘copers’^35^.

The limitation of this study is the recall bias of the history of previous ankle injuries. The data on the previous injury was solely based on players’ information. The severity of injury, the time lost from training and matches, and the number of recurrent injuries were not included in the study. We propose further prospective cohort studies with a larger group of players and more detailed parameters such as footballers’ psychosocial factors, injury mechanism, injury severity, patient perception, and treatment received.

## Conclusions

During preseason screening, half of the footballers in this study reported previous history of ankle injury, putting them at risk of having future ankle injuries. Footballers with previous ankle injuries have significantly less ankle inversion compared to those without. Additionally, significant numbers of the previously injured group also demonstrated persistent laxity. There was no significant deficit in other range of motion, ankle stability index, or muscle strength between those who had previously been injured and those who had not. However, as injury causation is multifactorial, preventive measures should be taken to reduce the risk of having an injury during the competitive season.
